# Modelling the effects of optical vibrations on photon beam parameters using ray-tracing software^1^

**DOI:** 10.1107/S1600577521007013

**Published:** 2021-08-12

**Authors:** C. Houghton, C. Bloomer, L. Alianelli

**Affiliations:** a Diamond Light Source Ltd, United Kingdom

**Keywords:** synchrotron, X-rays, *OASYS*, simulations, shadows

## Abstract

A method to simulate beam properties observed at the beamline sample-point in the presence of motion of optical components has been developed at Diamond Light Source. A series of stationary ray-tracing simulations are used to model the impact on the beam stability caused by dynamic motion of optical elements. Ray-tracing simulations using *SHADOW3* in *OASYS*, completed over multiple iterations and stitched together, permit the modelling of a pseudo-dynamic beamline. As beamline detectors operating at higher frequencies become more common, beam stability is crucial. Synchrotron ring upgrades to low-emittance lattices require increased stability of beamlines in order to conserve beam brightness. By simulating the change in beam size and position, an estimate of the impact the motion of various components have on stability is possible. The results presented in this paper focus on modelling the physical vibration of optical elements. Multiple beam parameters can be analysed in succession without manual input. The simulation code is described and the initial results obtained are presented. This method can be applied during beamline design and operation for the identification of optical elements that may introduce large errors in the beam properties at the sample-point.

## Abstract 2

Simulation results for dynamic ray-tracing software are compared with experimental data showing the impact of vibrations on sample-point X-ray beams.

## 1. Introduction   

Beam motion can be broadly described in two ways: ‘fast’ or ‘slow’. ‘Fast’ motions have periodicity equal to or shorter than the integration period of the primary beamline detector and therefore cause blurring of focal spots, but no visible temporal variations. ‘Slow’ motions are resolved by the detector and are therefore observable as a variation in the photon beam intensity or position (Farvacque, 1998 ▸). Extensive research has been conducted into the stability of the electron beam, improving the emittance stability, orbit stability and source point motion as it passes through the insertion device (Rehm, 2012 ▸). However intensity changes are not solely due to the electron beam but also the X-ray beam as it propagates through the beamline. Each optical element used to manipulate the X-ray beam properties can introduce unwanted beam motion, independent of the electron beam. As beamline detectors operating at higher frequencies become more common, previous ‘fast’ motion vibrations are redefined as ‘slow’ and therefore need to be mitigated.

Without mitigation, the impact of mechanical vibrations could become the limiting factor for full exploration of new generation low-emittance synchrotron beams (Grizolli *et al.*, 2019 ▸). Current mitigation techniques can include feedback systems to correct X-ray beam drift, mechanical interventions to reduce the transmission of vibration from external sources through to the optical elements or installation of passive magnetic damping systems to damp vibrations entirely (Bloomer *et al.*, 2012 ▸; Yamazaki *et al.*, 2013 ▸; Diez-Jimenez *et al.*, 2019 ▸).

Such techniques are usually applied retrospectively in response to beam motion negatively impacting beamline experiments. However, if the biggest potential contributors to sample-point beam motion could be identified during the design phase of beamlines and their optics, the mitigation process could be expedited and preemptively reduce the worse case vibrations.

This paper describes a method for simulating the vibration of optical elements and their subsequent impact on beam properties. Commonly used ray-tracing software is used for the purposes of identifying which optical element has the largest impact on the photon beam at the sample-point. This knowledge helps beamline designers to concentrate their efforts on the most important optics, and to improve their stability in particular. This preemptive modelling work is essential to achieve the required beam size, position and intensity stability in next generation synchrotron beamlines.

## 2. Software   

Simulating the effects of vibrations on the sample-point can, in principle, be carried out mathematically using geometric formulae (Goto, 2015 ▸). However a limitation of these methods is that they do not take into account imperfections of the optics, and tend to assume an infinite acceptance aperture. Ray-tracing simulations are used to overcome these limitations. *OASYS* (Rebuffi & Sanchez del Rio, 2017 ▸) and *SHADOW3* (Sanchez del Rio *et al.*, 2011 ▸) are two of the publicly available codes used to model and optimize the optical layout of a beamline using ray-tracing. This allows the expected beam parameters at the sample-point to be assessed. *OASYS* is a graphical environment for modelling beamlines with functionality allowing for surface errors of mirrors and cystals to be considered. Simulations of a beamline take approximately 60 s for 1 000 000 rays.

The graphical user interface of *OASYS* was used to initially model the beamline. In *SHADOW*, all elements which interact with the beam (slits, monochromator, crystals, focusing mirrors) are referred to as optical elements (OEs). After modelling all of the necessary optical elements, *OASYS* can export the beamline model as a Python script. To understand the impact that a misalignment of each of the optical element has at the beamline sample-point, a series of manual iterations can be performed, changing the parameters of each element in turn.

We built our own Python interface to enable us to quickly edit and iterate through different *OASYS* model parameters within the exported Python script. For example, this enabled us to iterate through each optical element, and alter the pitch of each reflecting surface by a defined angle. The resulting X-ray beam profile at the sample-point is recorded. This quickly allows the elements that impact the sample-point beam position the most to be identified. Our code also enabled a linear ‘scan’ of the optical element angle or position to be conducted, recording the results at the sample-point at each scan step. At each step in the scan a Gaussian-distributed beam consisting of randomly generated rays is used as the source point. Finally, functionality was added to apply a sine wave to the optical element angle or offset instead of a linear scan. This function in particular is of great use in simulating the sample-point effects arising from a periodic vibration of different elements. The modelling of these ‘pseudo-vibrations’ in particular will be described in more detail.

**Figure 1:**
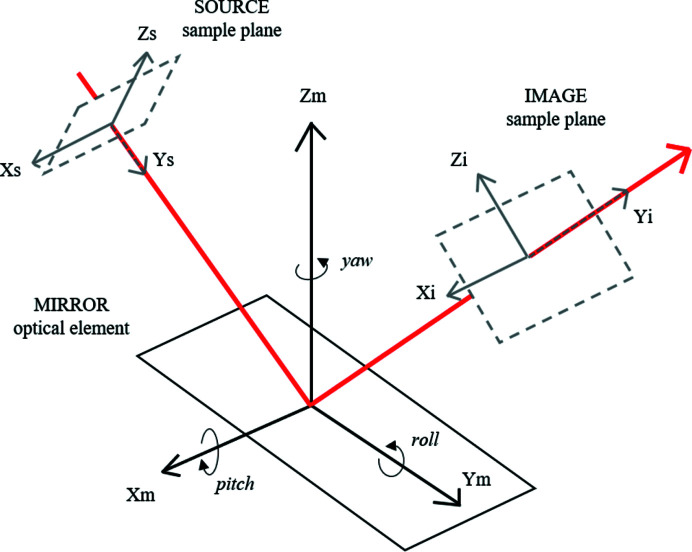
Reference frames used by *SHADOW* for the purposes of describing a mirror motion, as described in the *SHADOW* documentation.

Each optical element can be simulated to move in a number of ways. First, there are three transverse movements, which are simulated as sagittal, meridional and normal (*X*, *Y*, *Z*) offsets. Secondly, there are three angular motions of each optical element, with pitch, roll and yaw (*X*, *Y*, *Z*). These motions are illustrated in Fig. 1 ▸, as described by the *SHADOW* documentation. For each of these six parameters, our code carries out the sequence of ray-tracing simulations and recording each sample-point results, modelling the pseudo-vibrations.

For our initial work presented in this paper, each optical element parameter is treated independently. In reality, the overall motion of any optical element is a complex combination of motions along all possible axes, with coupling between transverse and angular movements. Additionally, in a real system different optical elements may oscillate totally independently; for example, two Kirkpatrick–Baez focusing mirrors mounted within a single vacuum vessel may oscillate in synchronicity due to some external source or resonance. Nonetheless, treating each optical element as an independent entity is useful to help determine which element has the biggest impact on the sample-point stability.

## 3. Data analysis   

To analyse the data obtained from the simulations, our code applied two processes: ‘percentage variation’ and ‘spectral analysis’. These are described below. Both methods provide valuable information that can be used to determine where to focus engineering efforts and will be discussed in turn.

### 3.1. Percentage variation   

Beam motion can be described in *absolute* terms, *i.e.* in real-world units of, say, micrometres; or it can be described in *relative* terms, for example given as a ‘proportion of beam size’, *i.e.* full width at half-maximum (FWHM). Of the two measures, it is typically more useful to refer to variations or misalignments in relative terms. For example, a beam mis­alignment at a sample-point measured to be 10 µm. Without the context of the beam size, this figure is not very meaningful. A 10 µm misalignment of a 1 mm beam is probably of little consequence; a 10 µm misalignment of a 1 µm beam may be disastrous. Thus, in our code we always report beam motion as a relative percentage variation: 10 µm misalignment of a 1 mm beam is 1%; 10 µm misalignment of a 1 µm beam is 10 000%.

Percentage variation analysis of each parameter is used to understand the change from the nominal starting value, which in this case is taken to be perfect alignment. Absolute values are less valuable when determining the impact of vibrations on the sample-point as beam stability is the main concern. When designing a beamline, it is also useful to express the desired beam stability in these percentage terms as well, as it is this relative change in beam parameters that best describes the potential impact on data collection. Beam motion is therefore always reported in this paper as a percentage of the beam size FWHM. Beam size variation is likewise always reported as a percentage of the FWHM.

For the arbitrary sample-point X-ray beam parameter *X*, the first data point, *X*_0_, corresponds to the unperturbed beam before any pseudo-vibration is applied. This is taken to be the zeroth point. Each subsequent iteration in the simulation applies a pitch or position offset to an optical element, modelling a pseudo vibration. The measured *X*_*i*_ is compared with the zeroth value and a percentage variation is calculated. We use the terms Δ*P*_*x*,*z*_ and Δσ_*x*,*z*_ to refer to the variation in beam position and beam size, respectively. Δ*I* is used to refer to the variation in beam intensity. 
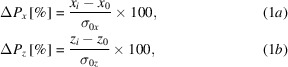


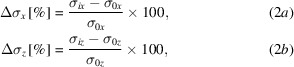


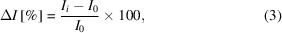
where Δ*P*_*x*,*z*_ is the beam displacement normalized to the beam width vertically (*x*) and horizontally (*z*) in accordance with the *SHADOW* reference frame, Δσ_*x*,*z*_ is the beam size in the vertical and horizontal, and Δ*I* is the intensity.

The variation of the beam position is compared with the FWHM of the beam size at the zeroth data point, σ_0 *x*,*z*_. This is done to place the data in context, as the impact of beam motion of 10 µm, for example, is different depending on the static ideal beam size for that particular experiment or beamline.

The r.m.s. sample-point variations in beam position, size and intensity following this iterative pseudo-vibration are recorded. Each optical element along the beam path has the same analysis performed by the code. The results of this analysis provide a clear indication of which optical element causes the largest variation of each of the ultimate sample-point X-ray beam parameters.

### 3.2. Spectral analysis   

Once the optical element that causes the largest variations in beam parameters has been identified, a more in-depth analysis of this variation is carried out. Since each step in the modelled pseudo-vibration represents a step in time, it is possible to perform a Fourier transform of the modelled sample-point beam position and intensity, and present the resulting beam parameters in both time and frequency domains.

The Fourier transform of the percentage variation data versus time is calculated, and the frequency at which the beam properties vary is determined from the location of the primary Fourier peak. For the purpose of our analysis, the vibration of an optical element with a given amplitude is assumed to result in the same sample-point impact irrespective of vibration frequency. All modelled pseudo-vibrations were sine waves with a frequency of 1 Hz; however, the ray-tracing results obtained at this frequency can be applied to other frequencies.

As the results in the following sections illustrate, this information is useful to help demonstrate how the vibration of an element at one frequency, *f*, can also result in sample-point variations at *harmonics* of this frequency, *e.g.* 2*f*, 3*f*, 4*f* and so on. For example, a monochromator crystal pitch vibration of frequency *f* could lead to intensity fluctuations at the sample of *2f* due to exceeding the width of the rocking curve.

## 4. Results   

### 4.1. Beamline overview   

**Figure 2:**
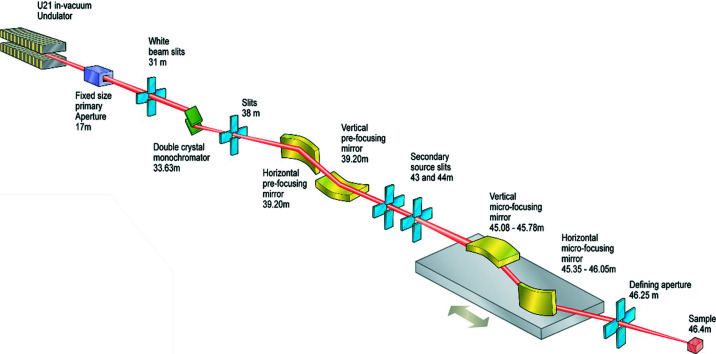
Schematic of Diamond Light Source Microfocus MX beamline I24 (Evans *et al.*, 2007 ▸).

The beamline chosen to evaluate the software performance was the Diamond Light Source Microfocus MX beamline I24 (Evans *et al.*, 2007 ▸). The optical layout is illustrated in Fig. 2 ▸. As this beamline uses a highly focused beam, high spatial resolution and flux stability within the focal spot size are important.

In the simulations completed for I24, a pseudo-vibration of the following elements was modelled: both crystals of Si(111) in the double crystal monochromator (DCM) separately (Crystal 1, Crystal 2), and four focusing mirrors consisting of two Kirkpatrick–Baez mirror systems (Suzuki & Uchida, 1991 ▸). The first mirror system containing the horizontal pre-focusing mirror (HPFM) and vertical pre-focusing mirror (VPFM) creates a virtual source point for the final focusing by the vertical micro-focusing mirror (VMFM) and the horizontal micro-focusing mirror (HMFM).

The beamline simulation presented in this paper is carried out using a modelled DCM energy of 12.4 keV, and a source divergence of 25.62 µrad horizontally and 4.48 µrad vertically. At the sample-point, under nominal conditions and with no pseudo-vibration applied, the modelled FWHM of the beam was 5.7 µm horizontally and 3.3 µm vertically.

A fixed-amplitude (1.0 µrad) pseudo-vibration is simulated for the axes of each optical element. The resulting r.m.s. amplitude of beam variation at the sample-point gives an excellent indication of the optical element that has the greatest influence on the ultimate beam stability. An example of this output is provided in Table 1 ▸, where the results of a ‘pitch’ variation of each optical element are presented, the pitch having generally the largest impact on the sample-point beam stability. From these results it becomes clearer that the largest r.m.s. beam motion at the sample-point was seen when a VPFM crystal pitch vibration was applied, closely followed by a vibration of either DCM crystal pitch. Additionally, it can be seen that a variation in the DCM crystal pitch produces the largest variations in intensity.

**Figure 3:**
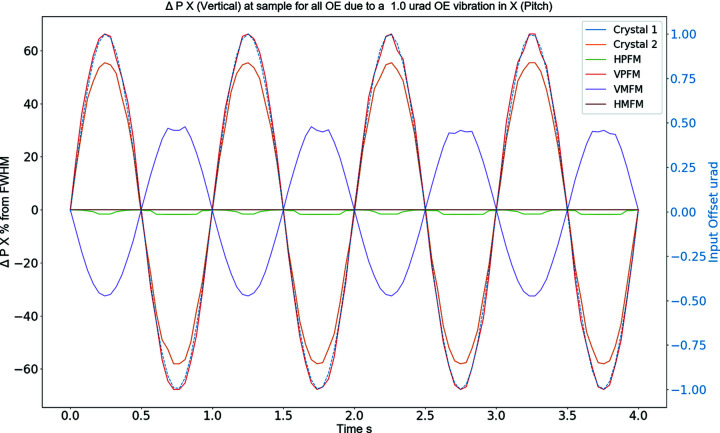
Graph showing the percentage variation of the vertical beam position at the sample-point due to a pitch vibration on each optical element.

Our code also plots the ‘time-series’ data from the pseudo-vibration simulation. This provides additional information that can help illustrate the effects that different optical elements can have at the sample-point. An example of these results is presented in Fig. 3 ▸ where the percentage variation of the beam position at the sample-point is plotted for a vibration of the pitch of each optical element.

The simulation produces data that are in-line with expected results. Variations in crystal pitch have been observed to have an adverse affect on the stability of sample-point intensity (Kristiansen *et al.*, 2015 ▸; Dolbnya *et al.*, 2019 ▸). Therefore, when investigating how pitch vibration impacts the beam at the sample-point, attention should be given to the DCM.

**Figure 4:**
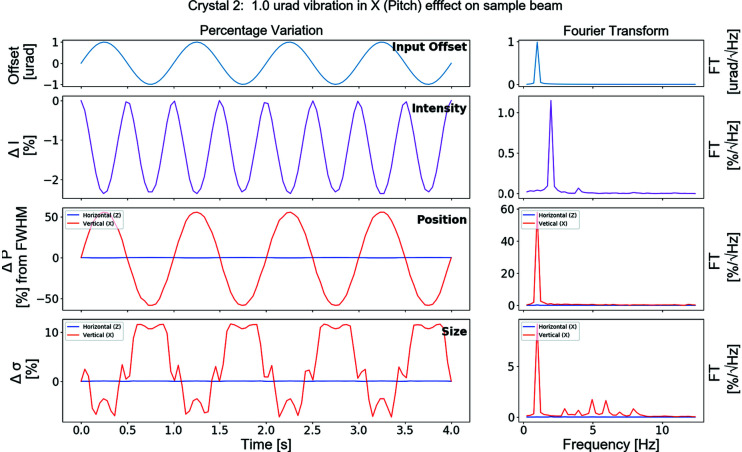
Graphs showing the results of the simulation of the second DCM crystal. Left: time series data. Right: frequency series data.

Further information regarding the expected sample-point stability can now be obtained by performing a spectral analysis of this time-series data. Presented in Fig. 4 ▸ is an example of the simulation results from this I24 model, plotting the results from a pseudo-vibration of the DCM second crystal pitch. The sample-point beam intensity, beam position and beam size are plotted.

The vibration applied to the pitch had a frequency of 1 Hz. This results in a 2 Hz intensity variation at the sample-point, with a smaller 4 Hz harmonic. This is shown in Fig. 4 ▸, second row.

As a micro-focus beamline the beam size and position stability are crucial. I24 has been designed for a beam size as small as 5 µm. The simulation shows that a 1 µrad vibration results in a 20% vertical beam size variation and vertical motion of 100% its initial FWHM (peak-to-peak). The standard beamline specification for stability is beam motion and beam size variation of less than 10% of the beam size. Thus, this simulation can be used to calculate a threshold of DCM crystal vibration which keeps the beam stability within the beamline specification.

### 4.2. Experimental data   

Simulation results were compared with experimental data obtained on the beamline. An angular pitch scan was applied to the second monochromator crystal, replicating the sine wave applied to the pitch in the simulations. The amplitude of the vibration applied was ±30 µrad. This was applied as a sinusoidal scan using a fine-pitch piezo motor. The intensity was measured using an X-ray beam position monitor located 0.16 m upstream of the sample-point. Beam size and position were measured using a fluorescence screen camera system placed at the sample-point. The camera images were fitted with a 2D Gaussian distribution to calculate the beam size, σ_*x*,*z*_, the beam position, and the centroid.

**Figure 5:**
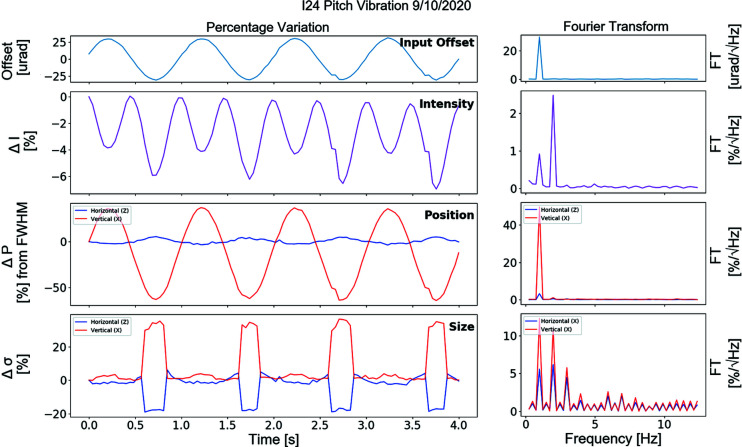
Graphs showing the experimental data results for the second DCM crystal angular change of ±30 µm.

As for the simulations these data were analysed using spectral analysis, and the results are shown in Fig. 5 ▸. The intensity data follow a similar trend to the simulation (Fig. 4 ▸) with a large peak at 2 Hz compared with the input 1 Hz motion. In addition, for both the position and the size data the overall trend appears to be consistent in that the vertical impact is larger than the horizontal impact.

The simulated data show intensity as double the input, with the 1 Hz peak being replaced with a 2 Hz peak. This is understood to arise due to the rocking curve of the DCM crystal. From an initial reflection angle at the peak of the rocking curve, any pitch change away from this point will decrease the intensity measured at the sample-point due to the beam moving off the rocking curve. Angular crystal changes in either direction will reduce the resulting beam intensity. In the plotted experimental data, the input vibration is not symmetrical around the peak of the rocking curve, which is likely due to starting the scan slightly away from the peak of the curve. The resultant intensity variation is therefore a combination of frequencies.

## 5. Discussion   

There is good qualitative agreement between the experimental data and simulation results. Quantitatively, there are inconsistencies in the percentage variation of all the beam parameters. The input crystal vibration amplitudes differed between the experiment and simulation. On the beamline, the crystal cannot be oscillated accurately to the level of ±1 µrad; however, the simulation has inaccuracies modelling larger angular crystal variations with the beam intensity decreasing by 96% with a ±30 µrad rotation. In addition, the experimental data show variation in the horizontal beam parameters not observed in the simulations. Real vibrations are more complex than simulated; there would be coupling between angles (*X* and *Z*). The disagreement between the model and experiment would decrease with a more accurate model for the beamline, in particular precise knowledge of slits and optics apertures, and the possible effects of crystal thermal bump on beam divergences. The simulation discussed in the paper does not include wave propagation through slits and the effects of diffraction. When slits are simulated, such as the secondary source slits, to beamline specifications there is little impact on the results. Surface imperfections on the mirrors cause higher frequency beam size variations in the experimental data as modelling these is challenging. Qualitative information is still valuable when designing beamlines as the frequency series results can help identify which optical elements produce harmonics when vibrated. In addition, the determination of which optical element causes the largest variation in beam parameters can direct efforts to stabilize movement before issues are noted by users.

Since this work was carried out, the authors note that some of these features have been incorporated into the latest release of *OASYS* (Rebuffi & Shi, 2020 ▸). For example, simple one-dimensional scanning of an optical element parameter (*e.g.* mirror pitch) can now be carried out using an implemented ‘Scanning Loops’ feature. This new addition will simplify the process of obtaining some of the results presented in this paper.

## 6. Conclusions   

We propose a method to perform dynamic modelling using *SHADOW* in *OASYS* similar to one recently incorporated in the software. In addition, we have introduced an original analysis of data, tailored for the typical optics instabilities observed on synchrotron beamline optics.

Minimization of optics instability is a major task in design and installation of elements such as crystals and (typically long) mirrors on synchrotron beamlines. Complex simulation of a full beamline as presented here will provide useful information to designers and engineers. Synchrotron source upgrades and lower emittance beams pose more stringent requirements on quality, alignment and precision of optical elements, thus understanding and eliminating dynamic effects such as vibrations is paramount. We have demonstrated a qualitative correlation between the impact of simulated pseudo vibration of optical elements and measured data.

## Figures and Tables

**Table 1:** The µrad input pitch vibration on each optical element

r.m.s. % variation	Δ*I*	Δσ_*z*_	Δσ_*x*_	Δ*P*_*z*_	Δ*P*_*x*_
Crystal 1	1.70	0.14	8.34	0.21	41.08
Crystal 2	1.70	0.05	8.27	0.21	41.08
HPFM	0.21	0.78	0.42	18.66	1.20
VPFM	0.07	0.00	7.99	0.05	47.86
VMFM	0.00	0.00	0.14	0.00	22.98
HMFM	0.00	0.14	0.00	9.62	0.00
